# Intuitionistic Linguistic Weighted Bonferroni Mean Operator and Its Application to Multiple Attribute Decision Making

**DOI:** 10.1155/2014/545049

**Published:** 2014-06-19

**Authors:** Peide Liu, Lili Rong, Yanchang Chu, Yanwei Li

**Affiliations:** ^1^School of Economics and Management, Civil Aviation University of China, Tianjin 300300, China; ^2^School of Management Science and Engineering, Shandong University of Finance and Economics, Jinan, Shandong 250014, China; ^3^School of Computer and Communications, Shandong TV University, Jinan, Shandong 250014, China

## Abstract

The intuitionistic linguistic variables are easier to describe the fuzzy information which widely exists in the real world, and Bonferroni mean can capture the interrelationship of the individual arguments. However, the traditional Bonferroni mean can only process the crisp number. In this paper, we will extend Bonferroni mean to the intuitionistic linguistic environment and propose a multiple attribute decision making method with intuitionistic linguistic information based on the extended Bonferroni mean which can consider the interrelationship of the attributes. Firstly, score function and accuracy function of intuitionistic linguistic numbers are introduced. Then, an intuitionistic linguistic Bonferroni mean (ILBM) operator and an intuitionistic linguistic weighted Bonferroni mean (ILWBM) operator are developed, and some desirable characteristics of them are studied. At the same time, some special cases with respect to the parameters *p* and *q* in Bonferroni are analyzed. Based on the ILWBM operator, the approach to multiple attribute decision making with intuitionistic linguistic information is proposed. Finally, an illustrative example is given to verify the developed approach and to demonstrate its effectiveness.

## 1. Introduction

Since the object things are fuzzy and uncertain, the attributes involved in the multiple attribute decision making (MADM) problems are not always expressed as crisp numbers, and some of them are more suitable to be denoted by fuzzy numbers, such as interval number, linguistic variable, and intuitionistic fuzzy number. Atanassov [1, 2] proposed the intuitionistic fuzzy set (IFS) characterized by a membership function and a nonmembership function, which is a generalization of the concept of fuzzy set proposed by Zadeh [3]. Later, Atanassov and Gargov [4] and Atanassov [5] further introduced the interval-valued intuitionistic fuzzy set (IVIFS), and Xu [6] and Wang [7] proposed the decision-making methods based on IVIFS. Zhang and Liu [8] defined the triangular intuitionistic fuzzy number, and they proposed the relevant decision making methods separately. Wang [9] gave the definition of intuitionistic trapezoidal fuzzy number and interval intuitionistic trapezoidal fuzzy number; then, some decision making methods based on the intuitionistic triangular fuzzy number had been proposed [10]. On the other hand, because linguistic variables are easy to express the qualitative information in evaluating the attributes, the decision making methods based on the linguistic variables have been a rapid development and a wide range of applications [11–14]. Furthermore, Wang and Li [15] proposed intuitionistic linguistic sets which combine intuitionistic fuzzy sets and linguistic variables, intuitionistic linguistic numbers, intuitionistic two-semantics, and the Hamming distance between two intuitionistic two-semantics and rank the alternatives by calculating the comprehensive membership degree to the ideal solution for each alternative. Obviously, intuitionistic linguistic sets are better to express the fuzzy information by integrating the advantages of intuitionistic fuzzy sets and linguistic variables, and they are receiving wide concerns. Liu [16] developed an intuitionistic linguistic generalized dependent ordered weighted average (ILGDOWA) operator and an intuitionistic linguistic generalized dependent hybrid weighted aggregation (ILGDHWA) operator. Liu and Wang [17] proposed an intuitionistic linguistic power generalized weighted average (ILPGWA) operator and an intuitionistic linguistic power generalized ordered weighted average (ILPGOWA) operator. On the basis of intuitionistic linguistic variables, Liu and Jin [18] further proposed the concept of intuitionistic uncertain linguistic variables (IULVs) and defined the operations on them, further developing some geometric average operators based on IULVs. Liu et al. [19] proposed the intuitionistic uncertain linguistic arithmetic Heronian mean (IULAHM) operator, intuitionistic uncertain linguistic weighted arithmetic Heronian mean (IULWAHM) operator, intuitionistic uncertain linguistic geometric Heronian mean (IULGHM) operator, and intuitionistic uncertain linguistic weighted geometric Heronian mean (IULWGHM) operator. Liu [20] proposed the concept of interval valued intuitionistic uncertain linguistic variables (IVIULVs) and defined the operations on them, further developing some geometric average operators based on IVIULVs. Obviously, now there are no researches on intuitionistic linguistic variables being applied to Bonferroni mean.

The information aggregation operators are an interesting and important research topic, which are receiving increasing concerns [16–28]. Bonferroni [22] originally proposed a Bonferroni mean (BM) operator, which has a desirable characteristic; that is, it can capture the expressed interrelationship of the individual arguments. Recently, Yager [23] further studied the BM and proposed an OWA variation of Bonferroni means, weighted Bonferroni aggregation, and Bonferroni choquet aggregation operator, and these generalizations enhance their modeling capability. Later, Beliakov et al. [24] proposed the generalized Bonferroni mean and discussed several interesting special cases with quite an intuitive interpretation for application. Xu and Yager [25] investigated the BM under intuitionistic fuzzy environments and developed an intuitionistic fuzzy BM (IFBM) and discussed its variety of special cases. Furthermore, they applied the weighted IFBM to multicriteria decision making and gave some numerical examples to illustrate related results. Beliakov and James [26] proposed the extending generalized Bonferroni means to Atanassov orthopairs in decision making contexts. Wei et al. [27] proposed the uncertain linguistic weighted Bonferroni mean operator (ULWBM) and the uncertain linguistic weighted geometric Bonferroni mean operator (ULWGBM). Liu and Jin [28] proposed some extended Bonferroni mean operators for trapezoid fuzzy linguistic variables, including a trapezoid fuzzy linguistic weighted Bonferroni mean operator (TFLWBM) and a trapezoid fuzzy linguistic weighted Bonferroni OWA operator (TFLWBOWA). Obviously, Bonferroni mean had been extended to intuitionistic fuzzy sets, uncertain linguistic variables, and trapezoid fuzzy linguistic variables. However, now Bonferroni mean has not been extended to intuitionistic linguistic variables.

The intuitionistic linguistic variables are very suitable to be used for depicting uncertain or fuzzy information, and Bonferroni mean can capture the interrelationship of the individual arguments. Motivated by the idea of IFBM operator proposed by Xu and Yager [25], this paper is to propose some Bonferroni operators, such as an intuitionistic linguistic Bonferroni mean (ILBM) operator and an intuitionistic linguistic weighted Bonferroni mean (ILWBM) operator, and some desirable properties of these operators are studied. At the same time, some special cases in these operators are analyzed. Based on the ILWBM operator, the approach to multiple attribute decision making with intuitionistic linguistic information is proposed.

The remainder of this paper is organized as follows. In Section 1, we give an introduction of the research background.  Section 2 briefly reviews some basic concepts and operations related to the intuitionistic linguistic variables and BM. In Section 3, an intuitionistic linguistic BM (ILBM) and an intuitionistic linguistic weighted BM (ILWBM) are developed, and some special cases are discussed.  Section 4 introduces a procedure for multiple attribute decision making based on ILWBM operator.  Section 5 gives an example to illustrate the decision making steps based on the proposed method and to analyze the affect on the decision-making results of the different parameters.  Section 6 ends this paper with some concluding remarks.

## 2. Preliminaries

### 2.1. The Linguistic Set and Its Extension

Suppose that *S* = (*s*_0_, *s*_1_,…, *s*_*l*−1_) is a finite and totally ordered discrete term set, where *l* is the odd value. In real situation, *l* is equal to 3, 5, 7, 9, and so forth. For example, when *l* = 9, a set *S* could be given as follows: *S* = (*s*_0_, *s*_1_, *s*_2_, *s*_3_, *s*_4_, *s*_5_, *s*_6_, *s*_7_, *s*_8_) = {extremely poor, very poor, poor, slightly poor, fair, slightly good, good, very good, extremely good}.

Usually, for any linguistic set *S*, it is required that *s*_*i*_ and *s*_*j*_ must satisfy the following additional characteristics [11, 12].

1. The set is ordered: *s*_*i*_≺*s*_*j*_, if and only if *i* < *j*.
2. There is the negation operator: neg(*s*_*i*_) = *s*_*l*−1−*i*_.
3. Maximum operator: max⁡(*s*_*i*_, *s*_*j*_) = *s*_*i*_, if *i* ≥ *j*.
4. Minimum operator: min⁡(*s*_*i*_, *s*_*j*_) = *s*_*i*_, if *i* ≤ *j*.

Furthermore, in order to preserve all the given information, Herrera et al. [11] proposed that the discrete linguistic label *S* = (*s*_0_, *s*_1_,…, *s*_*l*−1_) is extended to a continuous linguistic label $\overset{-}{S}=\{s_{\alpha }|\alpha \in R^{+}\}$ which satisfied the above characteristics.

For any linguistic variables $s_{i},s_{j}\in \overset{-}{S}$, the operations are defined as follows [13, 14]:

(1) $$\begin{array}{c} \beta s_{i}=s_{\beta \times i},\;\beta \geq 0,\\ s_{i}⊕s_{j}=s_{i+j},\\ \frac{s_{i}}{s_{j}}=s_{i/j},\;j!=0,\\ {\left(s_{i}\right)}^{n}=s_{i^{n}},\;n\geq 0. \end{array}$$

### 2.2. The Intuitionistic Linguistic Set (ILS)

**Definition 1 (see [15]).**

An ILS *A* in *X* is defined as

(2) $$\begin{array}{c} A=\left\{\langle x\left[h_{\theta (x)},\left(u_{A}\left(x\right),v_{A}\left(x\right)\right)\right]\rangle \;|\;x\in X\right\}, \end{array}$$

where $h_{\theta (x)}\in \overset{-}{S}$, *u*_*A*_ : *X* → [0,1], and *v*_*A*_ : *X* → [0,1], with the condition 0 ≤ *u*_*A*_(*x*) + *v*_*A*_(*x*) ≤ 1, for all *x* ∈ *X*. The numbers *u*_*A*_(*x*) and *v*_*A*_(*x*) represent, respectively, the membership degree and nonmembership degree of the element *x* to linguistic index *h*_*θ*(*x*)_.

For each ILS *A* in *X*, if *π*(*x*) = 1 − *u*_*A*_(*x*) − *v*_*A*_(*x*), for all *x* ∈ *X*, then *π*(*x*) is called a hesitancy degree of *x* to linguistic index *h*_*θ*(*x*)_. It is obvious that 0 ≤ *π*(*x*) ≤ 1, for all *x* ∈ *X*.

**Definition 2 (see [15]).**

Let *A* = {〈*x*[*h*_*θ*(*x*)_, (*u*_*A*_(*x*), *v*_*A*_(*x*))]〉∣*x* ∈ *X*} be ILS; the ternary group 〈*h*_*θ*(*x*)_, (*u*_*A*_(*x*), *v*_*A*_(*x*))〉 is called an intuitionistic linguistic number, and *A* can also be viewed as a collection of the intuitionistic linguistic number (ILN). So, it can also be expressed as *A* = {〈*h*_*θ*(*x*)_, (*u*_*A*_(*x*), *v*_*A*_(*x*))〉∣*x* ∈ *X*}. In addition, *π*_*A*_(*x*) = 1 − *u*_*A*_(*x*) − *v*_*A*_(*x*) represents the hesitancy degree, and it can also be called the intuitionistic linguistic fuzzy degree. For convenience, an ILN is denoted by $\tilde{a}=\langle s_{\theta (a)},(u(a),v(a))\rangle$, where *u*(*a*), *v*(*a*) ≥ 0, *u*(*a*) + *v*(*a*) ≤ 1.

Let ${\tilde{a}}_{1}=\langle s_{\theta (a_{1})},(u(a_{1}),v(a_{1}))\rangle$ and ${\tilde{a}}_{2}=\langle s_{\theta (a_{2})},(u(a_{2}),v(a_{2}))\rangle$ be two ILNs and *λ* ≥ 0; then, the operations of ILNs are defined as follows [15]:

(3) a~1+a~2=〈sθ(a1)+θ(a2),  (1−(1−u(a1))(1−u(a2)),v(a1)v(a2))〉,

(4) a~1a~2=〈sθ(a1)×θ(a2),  (u(a1)u(a2),v(a1)+v(a2)−v(a1)v(a2))〉,

(5) $$\begin{array}{c} \lambda {\tilde{a}}_{1}=\langle s_{\lambda \times \theta (a_{1})},\left(1-{\left(1-u\left(a_{1}\right)\right)}^{\lambda },{\left(v\left(a_{1}\right)\right)}^{\lambda }\right)\rangle , \end{array}$$

(6) $$\begin{array}{c} {{\tilde{a}}_{1}}^{\lambda }=\langle s_{{\left(\theta (a_{1})\right)}^{\lambda }},\left({\left(u\left(a_{1}\right)\right)}^{\lambda },1-{\left(1-v\left(a_{1}\right)\right)}^{\lambda }\right)\rangle . \end{array}$$

**Definition 3 (see [16]).**

Let ${\tilde{a}}_{1}=\langle s_{\theta (a_{1})},(u(a_{1}),v(a_{1}))\rangle$ be an ILN; a score function $S({\tilde{a}}_{1})$ of an ILN ${\tilde{a}}_{1}$ can be represented as follows:

(7) $$\begin{array}{c} S\left({\tilde{a}}_{1}\right)=\frac{\theta \left(a_{1}\right)}{l-1}\times \left[u\left(a_{1}\right)+\frac{1}{2}\left(1-u\left(a_{1}\right)-v\left(a_{1}\right)\right)\right]. \end{array}$$

**Definition 4 (see [16]).**

Let ${\tilde{a}}_{1}=\langle s_{\theta (a_{1})},(u(a_{1}),v(a_{1}))\rangle$ be an ILN; an accuracy function $H({\tilde{a}}_{1})$ of an ILN ${\tilde{a}}_{1}$ can be represented as follows:

(8) $$\begin{array}{c} H\left({\tilde{a}}_{1}\right)=\frac{\theta \left(a_{1}\right)}{l-1}\times \left(u\left(a_{1}\right)+v\left(a_{1}\right)\right). \end{array}$$

**Definition 5 (see [16]).**

If ${\tilde{a}}_{1}=\langle s_{\theta (a_{1})},(u(a_{1}),v(a_{1}))\rangle$ and ${\tilde{a}}_{2}=\langle s_{\theta (a_{2})},(u(a_{2}),v(a_{2}))\rangle$ are any two ILNs, then

1. if $S({\tilde{a}}_{1})>S({\tilde{a}}_{2})$, then ${\tilde{a}}_{1}≻{\tilde{a}}_{2}$;
2. if $S({\tilde{a}}_{1})=S({\tilde{a}}_{2})$, then if $H({\tilde{a}}_{1})>H({\tilde{a}}_{2})$, then ${\tilde{a}}_{1}≻{\tilde{a}}_{2}$; if $H({\tilde{a}}_{1})=H({\tilde{a}}_{2})$, then ${\tilde{a}}_{1}={\tilde{a}}_{2}$.

### 2.3. Bonferroni Mean (BM)

The BM was originally proposed by Bonferroni in [22], which was defined as follows.

**Definition 6 (see [22]).**

Let *p*, *q* ≥ 0, and let *a*_*i*_(*i* = 1,2,…, *n*) be a collection of nonnegative numbers. If

(9) $$\begin{array}{c} B^{p,q}\left(a_{1},a_{2},\ldots ,a_{n}\right)={\left(\frac{1}{n(n-1)}\overset{n}{\underset{\begin{array}{c} i,j=1\\ i\neq j \end{array}}{\sum }}a_{i}^{p}a_{j}^{q}\right)}^{1/(p+q)} \end{array}$$

then *B* ^*p*,*q*^ is called the Bonferroni mean (BM).

Obviously, the BM has the following properties.

1. *B* ^*p*,*q*^(0,0,…, 0) = 0.
2. *B* ^*p*,*q*^(*a*, *a*,…, *a*) = *a*, if *a*_*i*_ = *a*, for all *i*.
3. *B* ^*p*,*q*^(*a*_1_′, *a*_2_′,…, *a*_*n*_′) ≥ *B* ^*p*,*q*^(*a*_1_, *a*_2_,…, *a*_*n*_); that is, *B* ^*p*,*q*^ is monotonic, if *a*_*i*_′ ≥ *a*_*i*_, for all *i*.
4. min⁡_*i*_(*a*_*i*_) ≤ *B* ^*p*,*q*^(*a*_1_, *a*_2_,…, *a*_*n*_) ≤ max⁡_*i*_(*a*_*i*_).

If *p* = 1 and *q* = 1, then (9) reduces to the following:

(10) $$\begin{array}{c} B^{1,1}\left(a_{1},a_{2},\ldots ,a_{n}\right)={\left(\frac{1}{n(n-1)}\overset{n}{\underset{\begin{array}{c} i,j=1\\ i\neq j \end{array}}{\sum }}a_{i}a_{j}\right)}^{1/2}. \end{array}$$

If *q* = 0, (9) reduces to the following:

(11) Bp,0(a1,a2,…,an)=(1n(n−1)∑i,j=1i≠jnaipaj0)1/(p+0)=(1n∑i=1naip)1/p

which is a generalized mean operator; particularly the following cases hold.

(1) If *p* = 1 and *q* = 0, then (11) reduces to the usual average
  (12) $$\begin{array}{c} B^{1,0}\left(a_{1},a_{2},\ldots ,a_{n}\right)=\frac{1}{n}\overset{n}{\underset{i=1}{\sum }}a_{i}. \end{array}$$
(2) If *p* → 0 and *q* = 0, then (11) reduces to the geometric mean operator
  (13) $$\begin{array}{c} \underset{p\to 0}{\lim}B^{p,0}\left(a_{1},a_{2},\ldots ,a_{n}\right)={\left(\overset{n}{\underset{i=1}{\prod }}a_{i}\right)}^{1/n}. \end{array}$$

## 3. The Intuitionistic Linguistic Weighted Bonferroni Mean Operators

The Bonferroni mean (BM) has a significant advantage of capturing the interrelationship of the individual arguments; however, the traditional BM can only process the crisp numbers and cannot deal with intuitionistic linguistic. In this section, we will extend BM to deal with intuitionistic linguistic information and develop an intuitionistic linguistic Bonferroni mean (ILBM) operator and an intuitionistic linguistic weighted Bonferroni mean (ILWBM) operator. Further, we will discuss some desirable characteristics of them and some special cases with respect to the parameters *p* and *q* in Bonferroni.

**Definition 7 .**

Let ${\tilde{a}}_{j}=\langle s_{\theta_{j}},(u_{j},v_{j})\rangle \;\;(j=1,2,\ldots ,n)$ be a collection of ILNs, and ILB : Ω^*n*^ → Ω; if

(14) $$\begin{array}{c} \text{IL}{\text{B}}^{p,q}\left({\tilde{a}}_{1},{\tilde{a}}_{2},\ldots ,{\tilde{a}}_{n}\right)={\left(\frac{1}{n(n-1)}\overset{n}{\underset{\begin{array}{c} i,j=1\\ i\neq j \end{array}}{\sum }}{\tilde{a}}_{i}^{p}{\tilde{a}}_{j}^{q}\right)}^{1/(p+q)}, \end{array}$$

where Ω is the set of all intuitionistic linguistic numbers and for any *p*, *q* ≥ 0, then ILB^*p*,*q*^ is called the intuitionistic linguistic Bonferroni mean (ILB).

According to the operations of ILNs, we can get the following result.

**Theorem 8 .**

Let *p*, *q* ≥ 0, and let ${\tilde{a}}_{j}=\langle s_{\theta_{j}},(u_{j},v_{j})\rangle \;\;(j=1,2,\ldots ,n)$ be a collection of ILNs. Then, the aggregated result by formula (14) is also an ILN, and

(15) ILBp,q(a~1,a~2,…,a~n)=〈s((1/n(n−1))∑i,j=1, i≠jnθipθjq)1/(p+q),   ((1−(∏i,j=1i≠jn(1−uipujq))1/n(n−1))1/(p+q),    1−(1−(∏i,j=1i≠jn(1−(1−vi)p           ×(1−vj)q))1/n(n−1))1/(p+q))〉.

We use mathematical induction to prove this theorem shown as follows.

**Proof**

(1) Firstly, we need to prove that

(16) ∑i,j=1i≠jna~ipa~jq=〈s∑i,j=1, i≠jnθipθjq,   (1−(∏i,j=1i≠jn(1−uipujq)),    ∏i,j=1i≠jn(1−(1−vi)p(1−vj)q))〉.

By the operations of ILNs defined in (3)–(6), we have

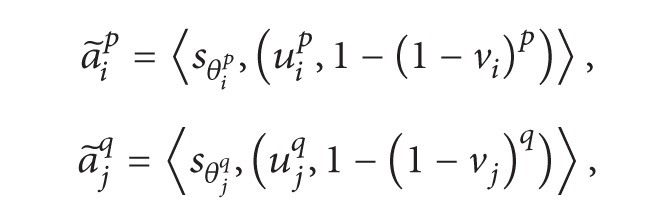
(17)

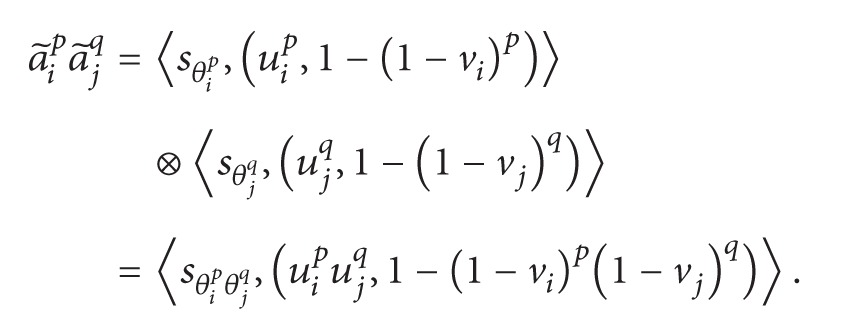
(18)

(a) When *n* = 2, by formulas (18) and (3), we can get

(19) ∑i,j=1i≠j2a~ipa~jq=a~1pa~2q+a~2pa~1q=〈sθ1pθ2q,(u1pu2q,1−(1−v1)p(1−v2)q)〉 +〈sθ2pθ1q,(u2pu1q,1−(1−v2)p(1−v1)q)〉=〈sθ1pθ2q+θ2pθ1q,(1−(1−u1pu2q)(1−u2pu1q),       (1−(1−v1)p(1−v2)q)       ×(1−(1−v2)p(1−v1)q))〉=〈s∑i,j=1, i≠j2θipθjq,  (1−∏i,j=1i≠j2(1−uipujq),   ∏i,j=1i≠j2(1−(1−vi)p(1−vj)q))〉;

that is, when *n* = 2, formula (16) is right.

(b) Suppose that when *n* = *k*, formula (16) is right; that is,

(20) ∑i,j=1i≠jka~ipa~jq=〈s∑i,j=1, i≠jkθipθjq,      (1−(∏i,j=1i≠jk(1−uipujq)),       ∏i,j=1i≠jk(1−(1−vi)p(1−vj)q))〉;

then, when *n* = *k* + 1, we have

(21) $$\begin{array}{c} \overset{k+1}{\underset{\begin{array}{c} i,j=1\\ i\neq j \end{array}}{\sum }}{\tilde{a}}_{i}^{p}{\tilde{a}}_{j}^{q}=\overset{k}{\underset{\begin{array}{c} i,j=1\\ i\neq j \end{array}}{\sum }}{\tilde{a}}_{i}^{p}{\tilde{a}}_{j}^{q}+\overset{k}{\underset{i=1}{\sum }}{\tilde{a}}_{i}^{p}{\tilde{a}}_{k+1}^{q}+\overset{k}{\underset{j=1}{\sum }}{\tilde{a}}_{k+1}^{p}{\tilde{a}}_{j}^{q}. \end{array}$$

Firstly, we prove that

(22) ∑i=1ka~ipa~k+1q =〈s∑i=1kθipθk+1q,   (1−∏i=1k(1−uipuk+1q),    ∏i=1k(1−(1−vi)p(1−vk+1)q))〉.

We also use the mathematical induction on *k* as follows.

(i) When *k* = 2, we have

(23) a~ipa~3q=〈sθipθ3q,(uipu3q,1−(1−vi)p(1−v3)q)〉∑i=12a~ipa~k+1q=a~1pa~3q+a~2pa~3q=〈sθ1pθ3q,(u1pu3q,1−(1−v1)p(1−v3)q)〉 +〈sθ2pθ3q,(u2pu3q,1−(1−v2)p(1−v3)q)〉=〈sθ1pθ3q+θ2pθ3q,(1−(1−u1pu3q)(1−u2pu3q),        (1−(1−v1)p(1−v3)q)        ×(1−(1−v2)p(1−v3)q))〉=〈s∑i=12θipθ3q,(1−∏i=12(1−uipu3q),        ∏i=12(1−(1−vi)p(1−v3)q))〉.

(ii) Suppose that *k* = *l*, then formula (22) is right; that is,

(24) ∑i=1la~ipa~l+1q=〈s∑i=1lθipθl+1q, (1−∏i=1l(1−uipul+1q),  ∏i=1l(1−(1−vi)p(1−vl+1)q))〉.

Then, when *k* = *l* + 1, we have

(25) ∑i=1l+1a~ipa~l+2q =∑i=1la~ipa~l+2q+a~l+1pa~l+2q =〈s∑i=1lθipθl+2q,   (1−∏i=1l(1−uipul+2q),    ∏i=1l(1−(1−vi)p(1−vl+2)q))〉  +〈sθl+1pθl+2q,(ul+1pul+2q,1−(1−vl+1)p(1−vl+2)q)〉 =〈s∑i=1l+1θipθl+2q,(1−∏i=1l+1(1−uipul+2q),          ∏i=1l+1(1−(1−vi)p(1−vl+2)q))〉;

that is, for *k* = *l* + 1, formula (22) is also right.

(iii) So, for all *k*, formula (22) is right.

Similarly, we can prove that

(26) ∑j=1ka~k+1pa~jq=〈s∑j=1kθk+1pθjq,  (1−∏j=1k(1−uk+1pujq),   ∏j=1k(1−(1−vk+1)p(1−vj)q))〉.

So, by formulas (20), (22), and (26), formula (21) can be transformed as

(27) ∑i,j=1i≠jk+1a~ipa~jq=∑i,j=1i≠jka~ipa~jq+∑i=1ka~ipa~k+1q+∑j=1ka~k+1pa~jq=〈s∑i,j=1, i≠jkθipθjq,  (1−(∏i,j=1i≠jk(1−uipujq)),    ∏i,j=1i≠jk(1−(1−vi)p(1−vj)q))〉 +〈s∑i=1kθipθk+1q,   (1−∏i=1k(1−uipuk+1q),    ∏i=1k(1−(1−vi)p(1−vk+1)q))〉 +〈s∑j=1kθk+1pθjq,   (1−∏j=1k(1−uk+1pujq),    ∏j=1k(1−(1−vk+1)p(1−vj)q))〉=〈s∑i,j=1, i≠jk+1θipθjq,   (1−(∏i,j=1i≠jk+1(1−uipujq)),    ∏i,j=1i≠jk+1(1−(1−vi)p(1−vj)q))〉.

So, when *n* = *k* + 1, formula (16) is also right.

Thus, formula (16) is right, for all *n*.

(2) Then, we can prove that formula (15) is right.

By formula (16), we can get

(28) ILBp,q(a~1,a~2,…,a~n)=(1n(n−1)∑i,j=1i≠jna~ipa~jq)1/(p+q)=(1n(n−1)〈s∑i,j=1, i≠jnθipθjq,          (1−(∏i,j=1i≠jn(1−uipujq)),            ∏i,j=1i≠jn(1−(1−vi)p(1−vj)q))〉)1/(p+q)=(〈s(1/(n(n−1)))∑i,j=1, i≠jnθipθjq,     (1−(∏i,j=1i≠jn(1−uipujq))1/n(n−1),       (∏i,j=1i≠jn(1−(1−vi)p        ×(1−vj)q))1/n(n−1))〉)1/(p+q)=〈s((1/n(n−1))∑i,j=1, i≠jnθipθjq)1/(p+q),   ((1−(∏i,j=1i≠jn(1−uipujq))1/n(n−1))1/(p+q),     1−(1−(∏i,j=1i≠jn(1−(1−vi)p           ×(1−vj)q))1/n(n−1))1/(p+q))〉.

**Example 9 .**

Suppose that there are three intuitionistic linguistic numbers ${\tilde{a}}_{1}=\langle s_{2},(0.6,0.1)\rangle$, ${\tilde{a}}_{2}=\langle s_{4},(0.4,0.3)\rangle$, and ${\tilde{a}}_{3}=\langle s_{1},(0.8,0.2)\rangle$, and suppose that *p* = 1 and *q* = 2; then, we can calculate the $\text{IL}{\text{B}}^{1,2}({\tilde{a}}_{1},{\tilde{a}}_{2},{\tilde{a}}_{3})$ shown as follows:

(29) ILB1,2(a~1,a~2,a~3)=〈s((1/(3×2))(θ1θ22+θ1θ32+θ2θ12+θ2θ32+θ3θ12+θ3θ22))1/(1+2),  ((1−((1−u1u22)(1−u1u32)      ×(1−u2u12)(1−u2u32)(1−u3u12)      ×(1−u3u22))1/(3×2))1/(1+2),   1−(1−(v−)1/(3×2))1/(1+2))〉,

where

(30) v−=(1−(1−v1)(1−v2)2)×(1−(1−v1)(1−v3)2)(1−(1−v2)(1−v1)2)×(1−(1−v2)(1−v3)2)(1−(1−v3)(1−v1)2)×(1−(1−v3)(1−v2)2).

Replace the data of ${\tilde{a}}_{1}$, ${\tilde{a}}_{2}$, and ${\tilde{a}}_{3}$; we can get

(31) v−=(1−(1−0.1)∗(1−0.3)2)∗(1−(1−0.1)∗(1−0.2)2)∗(1−(1−0.3)∗(1−0.1)2)∗(1−(1−0.3)∗(1−0.2)2)∗(1−(1−0.2)∗(1−0.1)2)∗(1−(1−0.2)∗(1−0.3)2)=  0.01212,ILB1,2(a~1,a~2,a~3)=〈s((1/(3×2))((2∗42+2∗12+4∗22+4∗12+1∗22+1∗42)))1/(1+2),  ((1−((1−0.6∗0.42)∗(1−0.6∗0.82)      ∗(1−0.4∗0.62)∗(1−0.4∗0.82)      ∗(1−0.8∗0.62)      ∗(1−0.8∗0.42))(1/6))(1/3),  1−(1−(0.01212)1/(3×2))1/(1+2))〉=〈s2.310,(0.606,0.195)〉.

In the following, we will discuss some special cases of the ILB^*p*,*q*^ operator shown as follows.

(1) When *q* = 0, formula (15) reduces to an intuitionistic linguistic generalized mean operator; it follows that

(32) ILBp,0(a~1,a~2,…,a~n) =〈s((1/n)∑i=1nθip)1/p,    ((1−(∏i=1n(1−uip))1/n)1/p,     1−(1−(∏i=1n(1−(1−vi)p))1/n)1/p)〉.

(2) If *p* = 1 and *q* = 0, then (15) reduces to an intuitionistic linguistic average operator

(33) B1,0(a1,a2,…,an)=1n∑i=1naiILB1,0(a~1,a~2,…,a~n) =〈s(1/n)∑i=1nθi,   ((1−(∏i=1n(1−ui))1/n),      (∏i=1nvi)1/n)〉.

(3) If *p* → 0 and *q* = 0, then (15) reduces to an intuitionistic linguistic geometric mean operator

(34) lim⁡p→0Bp,0(a1,a2,…,an) =〈s(∏i=1nθi)1/n,   ((∏i=1nui)1/n,1−(∏i=1n(1−vi))1/n)〉.

The traditional BM has the properties of commutativity, idempotency, monotonicity, and boundedness; in the following, we will prove that ILB also has these properties.

**Theorem 10 (commutativity).**

Let $\left({\tilde{a}}_{1}^{\prime },{\tilde{a}}_{2}^{\prime },\ldots ,{\tilde{a}}_{n}^{\prime }\right)$ be any permutation of $\left({\tilde{a}}_{1},{\tilde{a}}_{2},\ldots ,{\tilde{a}}_{n}\right)$; then,

(35) $$\begin{array}{c} ILB^{p,q}\left({\tilde{a}}_{1}^{\prime },{\tilde{a}}_{2}^{\prime },\ldots ,{\tilde{a}}_{n}^{\prime }\right)=ILB^{p,q}\left({\tilde{a}}_{1},{\tilde{a}}_{2},\ldots ,{\tilde{a}}_{n}\right). \end{array}$$

**Proof**

Let

(36) $$\begin{array}{c} {\text{ILB}}^{p,q}\left({\tilde{a}}_{1},{\tilde{a}}_{2},\ldots ,{\tilde{a}}_{n}\right)={\left(\frac{1}{n(n-1)}\overset{n}{\underset{\begin{array}{c} i,j=1\\ i\neq j \end{array}}{\sum }}{\tilde{a}}_{i}^{p}{\tilde{a}}_{j}^{q}\right)}^{1/(p+q)}\\ {\text{ILB}}^{p,q}\left({\tilde{a}}_{1}^{\prime },{\tilde{a}}_{2}^{\prime },\ldots ,{\tilde{a}}_{n}^{\prime }\right)={\left(\frac{1}{n(n-1)}\overset{n}{\underset{\begin{array}{c} i,j=1\\ i\neq j \end{array}}{\sum }}{\left({\tilde{a}}_{i}^{\prime }\right)}^{p}{\left({\tilde{a}}_{j}^{\prime }\right)}^{q}\right)}^{1/(p+q)}. \end{array}$$

Since $\left({\tilde{a}}_{1}^{\prime },{\tilde{a}}_{2}^{\prime },\ldots ,{\tilde{a}}_{n}^{\prime }\right)$ is any permutation of $\left({\tilde{a}}_{1},{\tilde{a}}_{2},\ldots ,{\tilde{a}}_{n}\right)$, we have

(37) (1n(n−1)∑i,j=1i≠jna~ipa~jq)1/(p+q) =(1n(n−1)∑i,j=1i≠jn(a~i′)p(a~j′)q)1/(p+q);

thus,

(38) $$\begin{array}{c} \text{IL}{\text{B}}^{p,q}\left({\tilde{a}}_{1}^{\prime },{\tilde{a}}_{2}^{\prime },\ldots ,{\tilde{a}}_{n}^{\prime }\right)=\text{IL}{\text{B}}^{p,q}\left({\tilde{a}}_{1},{\tilde{a}}_{2},\ldots ,{\tilde{a}}_{n}\right). \end{array}$$

**Theorem 11 (idempotency).**

Let ${\tilde{a}}_{j}=\tilde{a}$, *j* = 1,2,…, *n*; then $ILB^{p,q}({\tilde{a}}_{1},{\tilde{a}}_{2},\ldots ,{\tilde{a}}_{n})=\tilde{a}$.

**Proof**

Since ${\tilde{a}}_{j}=\tilde{a}$, for all *j*, we have

(39) ILBp,q(a~1,a~2,…,a~n)=(1n(n−1)∑i,j=1i≠jna~ipa~jq)1/(p+q)=(1n(n−1)∑i,j=1i≠jna~p+q)1/(p+q)=(a~p+q)1/(p+q)=a~.

**Theorem 12 (monotonicity).**

Let ${\tilde{a}}_{i}$(*i* = 1,2,…, *n*) and ${\tilde{b}}_{i}$(*i* = 1,2,…, *n*) be two collections of IFNs. If ${\tilde{a}}_{i}\geq {\tilde{b}}_{i}$, for all *i*, then

(40) $$\begin{array}{c} {ILB}^{p,q}\left({\tilde{a}}_{1},{\tilde{a}}_{2},\ldots ,{\tilde{a}}_{n}\right)\geq {ILB}^{p,q}\left({\tilde{b}}_{1},{\tilde{b}}_{2},\ldots ,{\tilde{b}}_{n}\right). \end{array}$$

**Proof**

Since ${\tilde{a}}_{i}\geq {\tilde{b}}_{i}$ for all *i*, we have

(41) $$\begin{array}{c} {\tilde{a}}_{i}^{p}{\tilde{a}}_{j}^{q}\geq {\tilde{b}}_{i}^{p}{\tilde{b}}_{j}^{q},\;\;\overset{n}{\underset{\begin{array}{c} i,j=1\\ i\neq j \end{array}}{\sum }}{\tilde{a}}_{i}^{p}{\tilde{a}}_{j}^{q}\geq \overset{n}{\underset{\begin{array}{c} i,j=1\\ i\neq j \end{array}}{\sum }}{\tilde{b}}_{i}^{p}{\tilde{b}}_{j}^{q}. \end{array}$$

So,

(42) $$\begin{array}{c} {\left(\frac{1}{n(n-1)}\overset{n}{\underset{\begin{array}{c} i,j=1\\ i\neq j \end{array}}{\sum }}{\tilde{a}}_{i}^{p}{\tilde{a}}_{j}^{q}\right)}^{1/(p+q)}\geq {\left(\frac{1}{n(n-1)}\overset{n}{\underset{\begin{array}{c} i,j=1\\ i\neq j \end{array}}{\sum }}{\tilde{b}}_{i}^{p}{\tilde{b}}_{j}^{q}\right)}^{1/(p+q)}; \end{array}$$

that is, $\text{IL}{\text{B}}^{p,q}({\tilde{a}}_{1},{\tilde{a}}_{2},\ldots ,{\tilde{a}}_{n})\geq \text{IL}{\text{B}}^{p,q}({\tilde{b}}_{1},{\tilde{b}}_{2},\ldots ,{\tilde{b}}_{n})$.

**Theorem 13 (boundedness).**

The *ILB* ^*p*,*q*^ operator lies between the max⁡ and min⁡ operators:

(43) min⁡(a~1,a~2,…,a~n)≤ILBp,q(a~1,a~2,…,a~n)≤max⁡(a~1,a~2,…,a~n).

**Proof**

Let $\tilde{a}=\min({\tilde{a}}_{1},{\tilde{a}}_{2},\ldots ,{\tilde{a}}_{n})$ and $\tilde{b}=\max({\tilde{a}}_{1},{\tilde{a}}_{2},\ldots ,{\tilde{a}}_{n})$.

Since $\tilde{a}\leq {\tilde{a}}_{j}\leq \tilde{b}$, then

(44) (1n(n−1)∑i,j=1i≠jna~pa~q)1/(p+q)≤(1n(n−1)∑i,j=1i≠jna~ipa~jq)1/(p+q)≤(1n(n−1)∑i,j=1i≠jnb~pb~q)1/(p+q).

That is,

(45) $$\begin{array}{c} \tilde{a}\leq {\left(\frac{\sum_{j=1}^{n}s\left({\tilde{a}}_{j},\tilde{x}\right){\tilde{a}}_{j}^{\lambda }}{\sum_{j=1}^{n}s\left({\tilde{a}}_{j},\tilde{x}\right)}\right)}^{1/\lambda }\leq \tilde{b}; \end{array}$$

that is, $\min({\tilde{a}}_{1},{\tilde{a}}_{2},\ldots ,{\tilde{a}}_{n})\leq \text{IL}{\text{B}}^{p,q}\leq \max({\tilde{a}}_{1},{\tilde{a}}_{2},\ldots ,{\tilde{a}}_{n})$.

In ILB^*p*,*q*^ operator, we only consider the input parameters and their interrelationships and do not consider the importance of each input parameter itself. However, in many practical situations, the weight of input data is also an important parameter. So, we can define an intuitionistic linguistic weighted Bonferroni mean (ILWBM) operator.

**Definition 14 .**

Let ${\tilde{a}}_{j}=\langle s_{\theta ({\tilde{a}}_{j})},(u({\tilde{a}}_{j}),v({\tilde{a}}_{j}))\rangle \;\;(j=1,2,\ldots ,n)$ be a collection of ILNs, and ILWB : Ω^*n*^ → Ω, if

(46) $$\begin{array}{c} {\text{ILWB}}_{\omega }^{p,q}\left({\tilde{a}}_{1},{\tilde{a}}_{2},\ldots ,{\tilde{a}}_{n}\right)={\left(\frac{1}{n(n-1)}\overset{n}{\underset{\begin{array}{c} i,j=1\\ i\neq j \end{array}}{\sum }}{\tilde{b}}_{i}^{p}{\tilde{b}}_{j}^{q}\right)}^{1/(p+q)}, \end{array}$$

where Ω is the set of all intuitionistic linguistic numbers and *ω* = (*ω*_1_,*ω*_2_,…,*ω*_*n*_)^*T*^ is the weight vector of ${\tilde{a}}_{j}\;\;(j=1,2,\ldots ,n)$, *ω*_*j*_ ∈ [0,1], ∑_*j*=1_^*n*^ *ω*_*j*_ = 1. ${\tilde{b}}_{j}=n\omega_{j}{\tilde{a}}_{j}$; *n* is a balance parameter. Then, ILWB is called the intuitionistic linguistic weighted Bonferroni mean (ILWBM) operator.

**Theorem 15 .**

ILB operator is a special case of the ILWB operator.

**Proof**

If *ω* = (1/*n*,1/*n*,…,1/*n*)^*T*^, then ${\tilde{b}}_{j}=n\omega_{j}{\tilde{a}}_{j}=n\left(1/n\right){\tilde{a}}_{j}={\tilde{a}}_{j}$:

(47) ILWBωp,q(a~1,a~2,…,a~n) =(1n(n−1)∑i,j=1i≠jnb~ipb~jq)1/(p+q) =(1n(n−1)∑i,j=1i≠jna~ipa~jq)1/(p+q) =ILBp,q(a~1,a~2,…,a~n).

**Theorem 16 .**

Let *p*, *q* ≥ 0 and ${\tilde{a}}_{j}=\langle s_{\theta_{j}},(u_{j},v_{j})\rangle$(*j* = 1,2,…, *n*) be a collection of ILNs, and *ω* = (*ω*_1_,*ω*_2_,…,*ω*_*n*_)^*T*^ is the weight vector of ${\tilde{a}}_{j}\;\;(j=1,2,\ldots ,n)$, *ω*_*j*_ ∈ [0,1], ∑_*j*=1_^*n*^ *ω*_*j*_ = 1. Then, the aggregated result by formula (46) is also an ILN, and

(48) ILWBωp,q(a~1,a~2,…,a~n)=〈s((np+q−1/(n−1))∑i,j=1, i≠jnωipωjqθipθjq)1/(p+q),  ((1−(∏i,j=1i≠jn(1−(1−(1−ui)nωi)p         ×(1−(1−uj)nωj)q))1/n(n−1))1/(p+q),    1−(1−(∏i,j=1i≠jn(1−(1−vinωi)p           ×(1−vjnωj)q))1/n(n−1))1/(p+q))〉.

Similar to Theorem 8, it can be proved by using mathematical induction on *n*.

**Example 17 .**

Suppose that there are three intuitionistic linguistic numbers ${\tilde{a}}_{1}=\langle s_{2},(0.6,0.1)\rangle$, ${\tilde{a}}_{2}=\langle s_{4},(0.4,0.3)\rangle$, and ${\tilde{a}}_{3}=\langle s_{1},(0.8,0.2)\rangle$, and the weight vector *ω* = (0.40,0.35,0.25) (suppose *p* = 1 and *q* = 2); then, we can calculate the $\text{ILW}{\text{B}}^{1,2}({\tilde{a}}_{1},{\tilde{a}}_{2},{\tilde{a}}_{3})$ shown as follows:

(49) ILWB1,2(a~1,a~2,a~3) =〈s((31+2−1/(3−1))(θ−))1/(1+2),   ((1−(u−)1/(3×2))1/(1+2),    1−(1−(v−)1/(3×2))1/(1+2))〉,

where

(50) θ−=ω1ω22θ1θ22+ω1ω32θ1θ32+ω2ω12θ2θ12+ω2ω32θ2θ32+ω31ω12θ3θ12+ω31ω22θ3θ22,u−=∏i,j=1i≠j3(1−(1−(1−ui)3ωi)(1−(1−uj)3ωj)2)=(1−(1−(1−u1)3∗ω1)∗(1−(1−u2)3∗ω2)2) ∗(1−(1−(1−u1)3∗ω1)∗(1−(1−u3)3∗ω3)2) ∗(1−(1−(1−u2)3∗ω2)∗(1−(1−u1)3∗ω1)2) ∗(1−(1−(1−u2)3∗ω2)∗(1−(1−u3)3∗ω3)2) ∗(1−(1−(1−u3)3∗ω3)∗(1−(1−u1)3∗ω1)2) ∗(1−(1−(1−u3)3∗ω3)∗(1−(1−u2)3∗ω2)2),v−=∏i,j=1i≠j3(1−(1−vi3ωi)(1−vj3ωj)2)=(1−(1−v13∗ω1)∗(1−v23∗ω2)2) ∗(1−(1−v13∗ω1)∗(1−v33∗ω3)2) ∗(1−(1−v23∗ω2)∗(1−v13∗ω1)2) ∗(1−(1−v23∗ω2)∗(1−v33∗ω3)2) ∗(1−(1−v33∗ω3)∗(1−v13∗ω1)2) ∗(1−(1−v33∗ω3)∗(1−v23∗ω2)2).

Replace the data of ${\tilde{a}}_{1}$, ${\tilde{a}}_{2}$, and ${\tilde{a}}_{3}$; we can get

(51) θ−=0.4∗0.352∗2∗42+0.4∗0.25∗2 ∗12+0.35∗0.42∗4∗22+0.35 ∗0.252∗4∗12+0.25∗0.42∗1∗22 +0.25∗0.352∗1∗42=3.4015,u−=(1−(1−(1−0.6)(3∗0.4))  ∗(1−(1−0.4)(3∗0.35))2) ∗(1−(1−(1−0.6)(3∗0.4))  ∗(1−(1−0.8)(3∗0.25))2) ∗(1−(1−(1−0.4)(3∗0.35))  ∗(1−(1−0.6)(3∗0.4))2) ∗(1−(1−(1−0.4)(3∗0.35))  ∗(1−(1−0.8)(3∗0.25))2) ∗(1−(1−(1−0.8)(3∗0.25))∗(1−(1−0.6)(3∗0.4))2) ∗(1−(1−(1−0.8)(3∗0.25))∗(1−(1−0.4)(3∗0.35))2)=0.23367,v−=(1−(1−0.1(3∗0.4))∗(1−0.3(3∗0.35))2) ∗(1−(1−0.1(3∗0.4))∗(1−0.2(3∗0.25))2) ∗(1−(1−0.3(3∗0.35))∗(1−0.1(3∗0.4))2) ∗(1−(1−0.3(3∗0.35))∗(1−0.2(3∗0.25))2) ∗(1−(1−0.2(3∗0.25))∗(1−0.1(3∗0.4))2) ∗(1−(1−0.2(3∗0.25))∗(1−0.3(3∗0.35))2)=0.01646;

then,

(52) ILWB1,2(a~1,a~2,a~3) =〈s((31+2−1/(3−1))(θ−))1/(1+2),   ((1−(u−)1/(3×2))1/(1+2),    1−(1−(v−)1/(3×2))1/(1+2))〉 =〈s2.4829,(0.5992,0.2086)〉.

It is easy to prove that the ILWB^*p*,*q*^ operator hasthe properties of commutativity and monotonicity, but it has not the property of idempotency.

## 4. An Approach to Multiple Attribute Decision Making Based on the Intuitionistic Linguistic Numbers

In the previous section, we extended BM to intuitionistic linguistic information and proposed ILBM and ILWBM operators. In this part, we will apply these extended BM operators to solve the multiple attribute decision making problems with intuitionistic linguistic information and give the detail decision making steps. The advantage of the proposed method is that it can consider the interrelationship of the attributes.

Consider a multiple attribute decision making with intuitionistic linguistic information: let *A* = {*A*_1_, *A*_2_,…, *A*_*m*_} be a discrete set of alternatives, and let *C* = {*C*_1_, *C*_2_,…, *C*_*n*_} be the set of attributes; *ω* = (*ω*_1_, *ω*_2_,…, *ω*_*n*_)^*T*^ is the weighting vector of the attribute *C*_*j*_(*j* = 1,2,…, *n*), where *ω*_*j*_ ≥ 0, *j* = 1,2,…, *n*, ∑_*j*=1_^*n*^ *ω*_*j*_ = 1. Suppose that $\tilde{X}={\left[{\tilde{x}}_{ij}\right]}_{m\times n}$ is the decision matrix, where ${\tilde{x}}_{ij}=\langle s_{a_{ij}},\left(u_{ij},v_{ij}\right)\rangle$ takes the form of the intuitionistic linguistic number, and 0 ≤ *u*_*ij*_ ≤ 1, 0 ≤ *v*_*ij*_ ≤ 1, *u*_*ij*_ + *v*_*ij*_ ≤ 1, *s*_*a*_*ij*__ ∈ *S*. Then, the ranking of alternatives is required.

In the following, we apply ILWB^*p*,*q*^ operator to multiple attribute decision making based on intuitionistic linguistic information.

The methods involve the following steps.

**Step 1 (normalization).**

Generally, there are two attribute types in multiple attribute decision making: they are benefit type (the bigger the performance values the better) and cost type (the smaller the performance values the better); we need normalization in order to transform the performance values of the cost type into the performance values of the benefit type. Then, $\tilde{X}={\left[{\tilde{x}}_{ij}\right]}_{m\times n}$ will be transformed into the matrix $\tilde{R}={\left[{\tilde{r}}_{ij}\right]}_{m\times n}$, where

1. ${\tilde{r}}_{ij}=\langle s_{a_{ij}},\left(u_{ij},v_{ij}\right)\rangle$, for benefit type of *C*_*j*_;
2. ${\tilde{r}}_{ij}=\langle \text{Neg}(s_{a_{ij}}),\left(v_{ij},u_{ij}\right)\rangle$, for cost type of *C*_*j*_.

**Step 2 .**

Calculate the comprehensive evaluation values of each alternative by ILWB^*p*,*q*^ operator:

(53) r~i=〈sai,(ui,vi)〉=ILWBωp,q(r~i1,r~i2,…,r~in)=〈s((np+q−1/(n−1))∑k,j=1, k≠jnωkpωjqaikpaijq)1/(p+q),  ((1−(∏k,j=1k≠jn(1−(1−(1−uik)nωk)p         ×(1−(1−uij)nωj)q))1/n(n−1))1/(p+q),  1−(1−(∏k,j=1k≠jn(1−(1−viknωk)p          ×(1−vijnωj)q))1/n(n−1))1/(p+q))〉.

**Step 3 .**

Rank the intuitionistic linguistic number ${\tilde{r}}_{i}$ by Definition 5.

**Step 4 .**

Rank all the alternatives *A* = {*A*_1_, *A*_2_,…, *A*_*m*_} in accordance with ${\tilde{r}}_{i}$ in descending order, and then select the most desirable alternative with the largest overall performance value.

**Step 5 .**

End.

## 5. Example

Let us suppose an investment company, which wants to invest a sum of money in the best option. There is a panel with four possible alternatives in which to invest the money:

1. *A*_1_ is a car company;
2. *A*_2_ is a computer company;
3. *A*_3_ is a TV company;
4. *A*_4_ is a food company.

The investment company must make a decision according to the following four attributes (suppose that the weight vector of four attributes is *ω* = (0.32,0.26,0.18,0.24)^*T*^):

1. *C*_1_ is the risk analysis;
2. *C*_2_ is the growth analysis;
3. *C*_3_ is the social-political impact analysis;
4. *C*_4_ is the environmental impact analysis.

The four possible alternatives {*A*_1_, *A*_2_, *A*_3_, *A*_4_} are evaluated using the linguistic term set *S* = (*s*_0_, *s*_1_, *s*_2_, *s*_3_, *s*_4_, *s*_5_, *s*_6_) under the above four attributes, and the decision matrix $\tilde{X}={\left[{\tilde{x}}_{ij}\right]}_{4\times 4}$ is listed in Table 1.

### 5.1. Decision Steps

To get the best alternative(s), the following steps are involved.

**Step 1 (normalization).**

Because all the attributes *C*_1_, *C*_2_, *C*_3_, *C*_4_ are the benefit type, the decision making matrix $\tilde{X}$ does not need normalization.

**Step 2 .**

Calculate the comprehensive evaluation values of each alternative by ILWB^*p*,*q*^ operator (here let *p* = *q* = 1).

From formula (53), we can get

(54) $$\begin{array}{c} {\tilde{r}}_{1}=\langle s_{3.979},\left(0.670,0.212\right)\rangle ,\\ {\tilde{r}}_{2}=\langle s_{3.544},\left(0.644,0.250\right)\rangle ,\\ {\tilde{r}}_{3}=\langle s_{3.035},\left(0.667,0.153\right)\rangle ,\\ {\tilde{r}}_{4}=\langle s_{3.411},\left(0.568,0.229\right)\rangle . \end{array}$$

**Step 3 .**

Rank the intuitionistic linguistic number ${\tilde{r}}_{i}$ by Definition 5:

(55) $$\begin{array}{c} S\left({\tilde{r}}_{1}\right)=0.483,\;\;S\left({\tilde{r}}_{2}\right)=0.412,\\ S\left({\tilde{r}}_{3}\right)=0.383,\;\;S\left({\tilde{r}}_{4}\right)=0.381. \end{array}$$

**Step 4 .**

Rank all the alternatives *A* = {*A*_1_, *A*_2_, *A*_3_, *A*_4_} in accordance with ${\tilde{r}}_{i}$ in descending order; we can get

(56) $$\begin{array}{c} A_{1}≻A_{2}≻A_{3}≻A_{4}. \end{array}$$

### 5.2. Discussions

#### 5.2.1. About the Influence of the Parameters *p*, *q* on Decision Making Result

In order to illustrate the influence of the parameters *p*, *q* on decision making result of this example, we use the different values of *p*, *q* in Step 2 to rank the alternatives. The ranking results are shown in Table 2.

As we can see from Table 2, the ordering of the alternatives may be different for the different value *p*, *q* in ILWB^*p*,*q*^ operator. In general, we can take the values of the two parameters as *p* = *q* = 1, which is not only intuitive and simple but also considering the interrelationship of the attributes. In the special case where at least one of these two parameters takes the value of zero, the ILWB^*p*,*q*^ operator cannot capture the interrelationship of the individual arguments. Thus, the organization can properly select the desirable alternative according to his interest and the actual needs.

#### 5.2.2. About the Validity of This Method

In order to verify the validity of this method, we use the method proposed by Wang and Li [15], which is the first method to process the multiple attribute decision making problems with intuitionistic linguistic information; to rank this example, we can get the ranking as *A*_1_≻*A*_2_≻*A*_3_≻*A*_4_. Obviously, these two methods have the same ranking result when *p* = *q* = 1 or *p* = 1, *q* = 0.

#### 5.2.3. Comparing with the Existing Methods

There are many decision making methods to process the multiple attribute decision making problems in which the attribute value is crisp number. However, there are a few methods which can process the intuitionistic linguistic information.

The first method which can process intuitionistic linguistic information was proposed by Wang and Li [15], and it is based on the arithmetic weighted average operator of intuitionistic linguistic information. This method can be only a special case of ILWB^*p*,*q*^ when *p* = 1, *q* = 0 proposed in this paper. Obviously, the method in [15] cannot consider the interrelationship of the attributes. However, the proposed method in this paper can provide a generalized method with parameters *p* and *q* and can capture the interrelationship of the attributes.

Comparing with the methods proposed by Liu [16] and Liu and Wang [17], which can consider the relationships between the attributes and can process the intuitionistic linguistic information, however, these methods process the relationships between the attributes by adding a class of weighted vector; for example, in [16], the added weighted vector can be obtained by calculating the similarity of each attribute to mean value and in [17] the weighted vector is determined by the support degree between the attributes. Obviously, these methods consider the relationships between the attributes in an indirect way. However, the method proposed in this paper can directly calculate the relationship between attribute values, so we can call it interrelationship.

Based on above discussions, the proposed method has a significant advantage which can consider the interrelationship between two attribute values. Of course, there exist some shortcomings in the proposed method; for example, it only considered the interrelationship between two attributes and not for three attributes or more. In addition, this method is more complex in calculation than the other methods.

## 6. Conclusion

Bonferroni mean has a significant advantage which can capture the interrelationship of the individual arguments. However, the traditional Bonferroni mean can only process the crisp number. In this paper, we extended Bonferroni mean to the intuitionistic linguistic environment and proposed an intuitionistic linguistic Bonferroni mean (ILBM) operator and an intuitionistic linguistic weighted Bonferroni mean (ILWBM) operator. Furthermore, we discussed some desirable properties of these operators and analyzed their special cases. Based on the ILWBM operator, we proposed a multiple attribute decision making method with intuitionistic linguistic information which can not only consider the importance of each attribute but also reflect the interrelationship between the attributes. However, there exist some shortcomings in the proposed method; for example, it only considered the interrelationship between two attributes and not for three attributes or more. In addition, it is more complex in calculation than the other methods. In the future, we will extend ILWBM operator to process the interrelationship among three attributes, and not for three attributes, or extend BM operator to process the other fuzzy information. In addition, we will research the applications of the proposed operators to solve the real decision making problems.

## Figures and Tables

**Table 1:** Decision matrix $\tilde{X}$.

	*C*_1_	*C*_2_	*C*_3_	*C*_4_
*A*_1_	〈*s*_5_, (0.7, 0.1)〉	〈*s*_3_, (0.7, 0.3)〉	〈*s*_4_, (0.6, 0.2)〉	〈*s*_4_, (0.7, 0.2)〉
*A*_2_	〈*s*_4_, (0.6, 0.2)〉	〈*s*_5_, (0.6, 0.3)〉	〈*s*_2_, (0.8, 0.1)〉	〈*s*_3_, (0.6, 0.4)〉
*A*_3_	〈*s*_4_, (0.7, 0.2)〉	〈*s*_5_, (0.6, 0.2)〉	〈*s*_1_, (0.7, 0.1)〉	〈*s*_2_, (0.7, 0.1)〉
*A*_4_	〈*s*_3_, (0.5, 0.2)〉	〈*s*_3_, (0.7, 0.1)〉	〈*s*_4_, (0.6, 0.3)〉	〈*s*_4_, (0.5, 0.3)〉

**Table 2:** Ordering of the alternatives by utilizing the different *p*, *q* in ILWB^*p*,*q*^ operator.

*p*, *q*	Score function $S({\tilde{r}}_{i})\;\;\;(i=1,2,3,4)$	Ranking
$\begin{array}{c} p=1\\ q=1 \end{array}$	$\begin{array}{c} S({\tilde{r}}_{1})=0.483,S({\tilde{r}}_{2})=0.412\\ S({\tilde{r}}_{3})=0.383,S({\tilde{r}}_{4})=0.381 \end{array}$	*A*_1_≻*A*_2_≻*A*_3_≻*A*_4_

$\begin{array}{c} p=2\\ q=2 \end{array}$	$\begin{array}{c} S({\tilde{z}}_{1})=0.500,S({\tilde{z}}_{2})=0.445\\ S({\tilde{z}}_{3})=0.447,S({\tilde{z}}_{4})=0.385 \end{array}$	*A*_1_≻*A*_3_≻*A*_2_≻*A*_4_

$\begin{array}{c} p=1\\ q\to 0 \end{array}$	$\begin{array}{c} S({\tilde{z}}_{1})=0.510,S({\tilde{z}}_{2})=0.432\\ S({\tilde{z}}_{3})=0.412,S({\tilde{z}}_{4})=0.394 \end{array}$	*A*_1_≻*A*_2_≻*A*_3_≻*A*_4_

$\begin{array}{c} p\to 0\\ q\to 0 \end{array}$	$\begin{array}{c} S({\tilde{z}}_{1})=0.574,S({\tilde{z}}_{2})=0.472\\ S({\tilde{z}}_{3})=0.379,S({\tilde{z}}_{4})=0.499 \end{array}$	*A*_1_≻*A*_4_≻*A*_2_≻*A*_3_

$\begin{array}{c} p=0.5\\ q=0.5 \end{array}$	$\begin{array}{c} S({\tilde{z}}_{1})=0.475,S({\tilde{z}}_{2})=0.394\\ S({\tilde{z}}_{3})=0.347,S({\tilde{z}}_{4})=0.378 \end{array}$	*A*_1_≻*A*_2_≻*A*_4_≻*A*_3_

$\begin{array}{c} p=10\\ q=10 \end{array}$	$\begin{array}{c} S({\tilde{z}}_{1})=0.579,S({\tilde{z}}_{2})=0.565\\ S({\tilde{z}}_{3})=0.605,S({\tilde{z}}_{4})=0.423 \end{array}$	*A*_3_≻*A*_1_≻*A*_2_≻*A*_4_

## References

[1] Atanassov KT (1986). Intuitionistic fuzzy sets. *Fuzzy Sets and Systems*.

[2] Atanassov KT (1989). More on intuitionistic fuzzy sets. *Fuzzy Sets and Systems*.

[3] Zadeh LA (1965). Fuzzy sets. *Information and Control*.

[4] Atanassov K, Gargov G (1989). Interval valued intuitionistic fuzzy sets. *Fuzzy Sets and Systems*.

[5] Atanassov KT (1994). Operators over interval valued intuitionistic fuzzy sets. *Fuzzy Sets and Systems*.

[6] Xu ZS (2007). Models for multiple attribute decision making with intuitionistic fuzzy information. *International Journal of Uncertainty, Fuzziness and Knowlege-Based Systems*.

[7] Wang J-Q (2006). Multi-criteria interval intuitionistic fuzzy decision-making approach with incomplete certain information. *Control and Decision*.

[8] Zhang X, Liu P (2010). Method for aggregating triangular fuzzy intuitionistic fuzzy information and its application to decision making. *Technological and Economic Development of Economy*.

[9] Wang J-Q (2008). Overview on fuzzy multi-criteria decision-making approach. *Control and Decision*.

[10] Wang J-Q, Zhang Z (2008). Programming method of multi-criteria decision-making based on intuitionistic fuzzy number with incomplete certain information. *Control and Decision*.

[11] Herrera F, Herrera-Viedma E, Verdegay JL (1996). A model of consensus in group decision making under linguistic assessments. *Fuzzy Sets and Systems*.

[12] Herrera F, Herrera-Viedma E (2000). Linguistic decision analysis: steps for solving decision problems under linguistic information. *Fuzzy Sets and Systems*.

[13] Xu Z (2006). A note on linguistic hybrid arithmetic averaging operator in multiple attribute group decision making with linguistic information. *Group Decision and Negotiation*.

[14] Xu ZS (2006). Goal programming models for multiple attribute decision making under linguistic setting. *Journal of Management Sciences in China*.

[15] Wang JQ, Li JJ (2009). The multi-criteria group decision making method based on multi-granularity intuitionistic two semantics. *Science & Technology Information*.

[16] Liu PD (2013). Some generalized dependent aggregation operators with intuitionistic linguistic numbers and their application to group decision making. *Journal of Computer and System Sciences*.

[17] Liu PD, Wang YM (2014). Multiple attribute group decision making methods based on intuitionistic linguistic power generalized aggregation operators. *Applied Soft Computing*.

[18] Liu P, Jin F (2012). Methods for aggregating intuitionistic uncertain linguistic variables and their application to group decision making. *Information Sciences*.

[19] Liu PD, Liu ZM, Zhang X (2014). Some intuitionistic uncertain linguistic heronian mean operators and their application to group decision making. *Applied Mathematics and Computation*.

[20] Liu P (2013). Some geometric aggregation operators based on interval intuitionistic uncertain linguistic variables and their application to group decision making. *Applied Mathematical Modelling*.

[21] Liu PD (2014). Some Hamacher aggregation operators based on the interval-valued intuitionistic fuzzy numbers and their application to Group Decision Making. *IEEE Transactions on Fuzzy Systems*.

[22] Bonferroni C (1950). Sulle medie multiple di potenze. *Bolletino Matematica Italiana*.

[23] Yager RR (2009). On generalized Bonferroni mean operators for multi-criteria aggregation. *International Journal of Approximate Reasoning*.

[24] Beliakov G, James S, Mordelová J, Rückschlossová T, Yager RR (2010). Generalized Bonferroni mean operators in multi-criteria aggregation. *Fuzzy Sets and Systems*.

[25] Xu Z, Yager RR (2011). Intuitionistic fuzzy bonferroni means. *IEEE Transactions on Systems, Man, and Cybernetics B: Cybernetics*.

[26] Beliakov G, James S (2013). On extending generalized Bonferroni means to Atanassov orthopairs in decision making contexts. *Fuzzy Sets and Systems*.

[27] Wei G, Zhao X, Lin R, Wang H (2013). Uncertain linguistic Bonferroni mean operators and their application to multiple attribute decision making. *Applied Mathematical Modelling*.

[28] Liu PD, Jin F (2012). The trapezoid fuzzy linguistic Bonferroni mean operators and their application to multiple attribute decision making. *Scientia Iranica*.

